# Fitted computational method for solving singularly perturbed small time lag problem

**DOI:** 10.1186/s13104-022-06202-0

**Published:** 2022-10-11

**Authors:** Sisay Ketema Tesfaye, Mesfin Mekuria Woldaregay, Tekle Gemmechu Dinka, Gemechis File Duressa

**Affiliations:** 1 Department of Applied Mathematics, Adama Science and Technology University, Adama, Ethiopia; 2 Department of Mathematics, Jimma University, Jimma, Ethiopia

**Keywords:** Accurate numerical method, Exponentially fitted method, Stability and uniform convergence

## Abstract

**Objectives:**

An accurate exponentially fitted numerical method is developed to solve the singularly perturbed time lag problem. The solution to the problem exhibits a boundary layer as the perturbation parameter approaches zero. A priori bounds and properties of the continuous solution are discussed.

**Result:**

The backward-Euler method is applied in the time direction and the higher order finite difference method is employed for the spatial derivative approximation. An exponential fitting factor is induced on the difference scheme for stabilizing the computed solution. Using the comparison principle, the stability of the method is examined and analyzed. It is proved that the method converges uniformly with linear order of convergence. To validate the theoretical findings and analysis, two test examples are given. Comparison is made with the results available in the literature. The proposed method has better accuracy than the schemes in the literature.

## Introduction

A differential equation in which its highest order derivative term is multiplied by a small parameter is known as a singularly perturbed differential equation (SPDE). It commonly occurs in the modeling of chemical processes, fluid flows, water quality problems in river networks, mechanical systems and simulation of oil extraction from underground reservoirs [1]. The solution of such type of equation possesses a multi-scale character that varies quickly in the boundary layer region and it slows in the outer layer regions.

Due to the multi-scale character of the solution, the classical numerical methods fail to give an accurate result. Currently, it becomes interesting to develop a numerical method which gives accurate results; and its convergence does not depend on the perturbation parameter. For solving the considered problem in Kumar et al. [2] proposed an adaptive mesh method using the concept of entropy function. Hybrid scheme of the midpoint upwind in the outer region and the central difference method in the boundary layer region are used in [3, 4]. Gowrisankar and Natesan [5] used the upwind finite difference method on a piecewise uniform mesh. The upwind finite difference method on Shishkin mesh is used in [6]. Podila and Kumar [7] used a stable finite difference scheme, which works on a uniform and an adaptive mesh. An exponentially fitted scheme is discussed in [8, 9]. The non-standard finite difference method is used in [10]. The numerical schemes developed in [11–16] works for both the large and small delay cases.

In this paper, we proposed a numerical scheme using higher order finite difference scheme fitted by the exponential fitting factor. Moreover, the main aim of this study is to develop a more accurate, stable and uniformly convergent numerical scheme for solving singularly perturbed convection-diffusion problem having small time lag.

In this paper, *C* is a generic positive constant, which does not depend on the mesh parameters and the perturbation parameter. The norm $\Vert .\Vert$, defined by $\Vert g \Vert =max_{(s,t) \in \Omega }\vert g(s,t) |$ is the maximum norm.

## Continuous problem

Consider a class of singularly perturbed convection-diffusion problem of the form

1 $$\begin{aligned} \left\{ \begin{array}{lll} z_{t}(s,t) + {\mathcal {L}}z(s,t) = -c(s,t)z(s, t- \tau ) + g(s,t) , (s,t) \in \Omega =(0,1)\times (0,T], \\ z(s,t) = \psi _{b}(s,t) \ on \ \eta _{b}:= [0,1] \times [-\tau , 0], \\ z(0,t)= \psi _{l}(t) \ on \ \eta _{l} := \{ (0,t):0\le t\le T \}, \\ z(1,t) = \psi _{r}(t) \ on \ \eta _{r} := \{(1,t):0\le t \le T\} , \end{array}\right. \end{aligned}$$

where ${\mathcal {L}}z(s,t)=- \varepsilon z_{ss}(s,t) + b(s,t)z_{s}(s,t) + a(s)z(s,t)$, $0 < \varepsilon \ll 1$ is the perturbation parameter and $\tau > 0$ is the delay parameter. The coefficients $b(s,t),\ a(s), \ c(s,t), \ g(s,t)$ on $\bar{\Omega }$ and $\psi _{b}(s,t),\ \psi _{l}(t),\ \psi _{r}(t)$ on $\eta = \eta _{l}\cup \eta _{r} \cup \eta _{b}$ are assumed to be smooth and bounded functions that satisfy the conditions $a(s) \ge \alpha>0,\ c(s,t) \ge \gamma >0 , \ b(s,t) \le \beta <0 ,\ a(s)+ c(s,t)\ge 0$ on $\bar{\Omega }$. Under these conditions, the problem exhibits a boundary layer along $s=0$ [2].

### Properties of the analytical solution

In this part, we present the analytical aspects of the solution and its derivatives. The existence and uniqueness of a solution of (1).

#### Lemma 2.1

[17, 18]* The solution* *z*(*s*, *t*)* of* (1)* satisfies* $\vert z(s,t) - \psi _{b}(s,0)|\le Ct,\; (s,t)\in \bar{\Omega }=[0,1]\times [0,T],$* where* $C >0$* is a constant that does not depends on* $\varepsilon$.

The operator $L(s,t)= z_{t}(s,t) + {\mathcal {L}}z(s,t)$ of (1) satisfies the following minimum principle.

#### Lemma 2.2

[19]* Let* $\nu (s,t) \in C^{2}(\Omega ) \cap C^{0}(\bar{\Omega })$,* satisfies* $\nu (s,t) \ge 0$  $(s,t) \in \partial \Omega=\bar{\Omega}-\Omega$.* If* $L\nu (s,t) \le 0, ~(s,t) \in \Omega$*, then* $\nu (s,t) \ge 0, (s,t)\in \bar{\Omega }$.

#### Lemma 2.3

[18, 19]* [Stability result] The solution of (*1*) satisfies the bound*

2 $$\begin{aligned} \vert z(s,t)|\le \alpha ^{-1}\Vert g\Vert + \max \{\vert \psi _{l}(t)|, \vert \psi _{b}(s,t)\vert , \vert \phi _{r}(t)|\}, \end{aligned}$$

*where* $\alpha$ *is the lower bound of* *a*(*s*) *and* $\Vert g \Vert = \max _{s,t\in \Omega }\vert g(s,t)|$.

#### Lemma 2.4

[20, 21]* The following bounds are satisfied for the derivative of the solution* *z*(*s*, *t*)* of* (1)* with respect to* *s and* *t*

3 $$\begin{aligned} \bigg \vert \frac{\partial ^{n}z(s,t)}{\partial s^{n}}\bigg |\le C\left(1 + \varepsilon ^{-n}\exp \left(-\frac{\beta }{\varepsilon }s\right)\right), \ (s,t)\in \bar{\Omega }, \ n= 0,1,2,3,4, \end{aligned}$$

*and*

4 $$\begin{aligned} \bigg \vert \frac{\partial ^{l}z(s,t)}{\partial t^{l}}\bigg |\le C, \ (s,t) \in \bar{\Omega } , \ l = 0,1,2, \end{aligned}$$

*where* $\beta$* is lower bound of b*(*s*, *t*).

## Main text: numerical scheme

### Temporal semi-discretization

Using Taylor’s series expansion for the delay term; using a uniform mesh in *t*-direction with step size $\Delta t = T/M$ given by $\Omega _{t}^{M} = \{ t_{n} = n\Delta t, n = 0,1,2,...,M, t_{M} = T\}$, where *M* is the number of mesh points in [0, *T*]. Note that $T = r\tau$ for some positive integer *r*. Using the backward-Euler formula, we get

5 $$\begin{aligned} \begin{aligned} (1 - \tau c(s,t_{n}))\frac{z^{n}(s) - z^{n-1}(s)}{\Delta t}&-\varepsilon \frac{d^{2}z^{n}(s)}{ds^{2}} + b(s,t_{n})\frac{d z^{n}(s)}{ds} \\ {}&+(a(s)+c(s,t_{n}))z^{n}(s) = g(s,t_{n}). \end{aligned} \end{aligned}$$

Equivalently, we write

6 $$\begin{aligned} -\varepsilon \frac{\ d^{2}Z^{n}(s)}{\ ds^{2}} + b(s,t_{n})\frac{\ d Z^{n}(s)}{\ ds} + P(s,t_{n})Z^{n}(s) = R(s,t_{n}), \end{aligned}$$

where $P(s,t_{n}) = a(s)+c(s,t_{n})+\frac{1 - \tau c(s,t_{n})}{\Delta t},$ and $R(s,t_{n}) = g(s,t_{n})+ \frac{1 - \tau c(s,t_{n})}{\Delta t}Z^{n-1}(s)$ with the boundary

7 $$\begin{aligned} Z^{n}(0) = \psi _{l}(t_{n}),~~~~~~ Z^{n}(1) = \psi _{r}(t_{n}),~~~0\le n\le M. \end{aligned}$$

Now, we rewrite (6) as

8 $$\begin{aligned} -\varepsilon \frac{\ d^{2}Z(s)}{\ ds^{2}} + b(s,t_{n})\frac{\ dZ(s)}{\ d s} + P(s,t_{n})Z(s) = R(s,t_{n}), \end{aligned}$$

where $Z(s) = Z^{n}(s) = Z(s,t_{n})$. At each time step the local error is defined as $e_{n}(s): = z(s,t_{n}) - Z^{n}(s), \ 0\le n \le M.$

#### Lemma 3.1

*The local error estimate in the temporal direction satisfies the bound*

$$\begin{aligned} \Vert e_{n}\Vert \le C_{1}(\Delta t)^{2}, \end{aligned}$$

*and the global error at* $n^{th}$ *time level satisfies the bound*

9 $$\begin{aligned} \Vert E_{n}\Vert \le C(\Delta t). \end{aligned}$$

#### Proof

Refer from the Appendix section. $\square$

#### Lemma 3.2

*For* $0 \le n \le M-1$, *the solution of (*6)–(7), *satisfies the bound*

10 $$\begin{aligned} \bigg |\frac{d^{r}Z^{n}(s)}{ds^{r}} \bigg |\le C\left(1 + \varepsilon ^{-r}\exp \left(-\frac{\beta }{\varepsilon }(s)\right)\right),~~~s\in \bar{\Omega }_{s},~~0\le r\le 4. \end{aligned}$$

#### Proof

For the proof see [22]. $\square$

### Spatial discretization

The spatial domain [0, 1] into *N* equal number of sub-intervals with the length of *h* is given by $0= s_{0},s_{1},...,s_{N}=1$ and $s_{i} = ih, i = 0,1,2,...,N$. Assume smooth function $Z(s) = Z^{n}(s) = Z(s,t_{n})$ in the interval [0, 1]. From Taylor’s series expansion, we have

11 $$\begin{aligned} \begin{aligned} Z_{i+1} \approx\,&Z_{i}+hZ^{'}_{i}+\frac{h^{2}}{2!}Z^{''}_{i}+\frac{h^{3}}{3!}Z^{'''}_{i}+\frac{h^{4}}{4!}Z^{(4)}_{i} +\frac{h^{5}}{5!}Z^{(5)}_{i}+\frac{h^{6}}{6!}Z^{(6)}_{i}+\frac{h^{7}}{7!}Z^{(7)}_{i} +\frac{h^{8}}{8!}Z^{(8)}_{i},\\ Z_{i-1} \approx\,&Z_{i}-hZ^{'}_{i}+\frac{h^{2}}{2!}Z^{''}_{i}-\frac{h^{3}}{3!}Z^{'''}_{i}+\frac{h^{4}}{4!}Z^{(4)}_{i} -\frac{h^{5}}{5!}Z^{(5)}_{i}+\frac{h^{6}}{6!}Z^{(6)}_{i}-\frac{h^{7}}{7!}Z^{(7)}_{i} +\frac{h^{8}}{8!}Z^{(8)}_{i}. \end{aligned} \end{aligned}$$

Combining the result in (11), we arrive at

12 $$\begin{aligned} \begin{aligned} Z_{i-1} - 2Z_{i} + Z_{i+1} =&\frac{2h^{2}}{2!}Z^{''}_{i}+\frac{2h^{4}}{4!}Z^{(4)}_{i} +\frac{2h^{6}}{6!}Z^{(6)}_{i} +\frac{2h^{8}}{8!}Z^{(8)}_{i}+O(h^{10}) \\ Z^{''}_{i-1} - 2Z^{''}_{i} + Z^{''}_{i+1} =&\frac{2h^{2}}{2!}Z^{(4)}_{i}+\frac{2h^{4}}{4!}Z^{(6)}_{i} +\frac{2h^{6}}{6!}Z^{(8)}_{i} +\frac{2h^{8}}{8!}Z^{(10)}_{i}+O(h^{12}). \end{aligned} \end{aligned}$$

Using (12), we get $\frac{h^{4}}{12}Z^{(6)}_{i}$ and, we obtain

13 $$\begin{aligned} \begin{aligned} Z_{i-1} - 2Z_{i} + Z_{i+1} =&\frac{h^{2}}{30}(Z^{''}_{i-1}+28Z^{''}_{i} +Z^{''}_{i+1}) + \zeta , \end{aligned} \end{aligned}$$

where $\zeta = \frac{h^{4}}{20}Z^{(4)}_{i}- \frac{13h^{8}}{302,400}Z^{(8)}_{i} + O(h^{10})$. Using (8), we get

14 $$\begin{aligned} \begin{aligned} -\varepsilon Z^{''}_{i+1} =&-b(s_{i+1},t_{n})Z^{'}_{i+1} - P(s_{i+1},t_{n})Z_{i+1}+R(s_{i+1},t_{n}) ,\\ -\varepsilon Z^{''}_{i} =&-b(s_{i},t_{n})Z^{'}_{i} -P(s_{i},t_{n})Z_{i}+R(s_{i},t_{n}), \\ -\varepsilon Z^{''}_{i-1} =&-b(s_{i-1},t_{n})Z^{'}_{i-1} - P(s_{i-1},t_{n})Z_{i-1}+R(s_{i-1},t_{n}). \end{aligned} \end{aligned}$$

Next, using the non-symmetric finite difference schemes we approximate $Z^{'}_{i+1}, Z^{'}_{i}$ and $Z^{'}_{i+1}$ as

15 $$\begin{aligned} \begin{aligned} Z^{'}_{i} =&\frac{Z_{i+1} - Z_{i-1}}{2h}+O(h^2), \;\; Z^{'}_{i+1} = \frac{3Z_{i+1} -4Z_{i}+ Z_{i-1}}{2h} - hZ^{''}_{i}+O(h^{2}), \\ Z^{'}_{i-1} =&\frac{-Z_{i+1} +4Z_{i}- 3Z_{i-1}}{2h} + hZ^{''}_{i}+O(h^{2}). \end{aligned} \end{aligned}$$

Substituting (15) into (14) and substituting the resulting equation into (13) after simplifying, we obtain

16 $$\begin{aligned}&-{\left(\varepsilon -{\frac{hb(s_{i-1},t_{n})}{30}}+\frac{hb(s_{i+1},t_{n})}{30}\right)}\left({\frac{Z_{i-1} - 2Z_{i} + Z_{i+1}}{h^2}}\right) + {\frac{b(s_{i-1},t_{n})}{60h}}(-3Z_{i-1}+4Z_{i}-Z_{i+1})\\&+{\frac{28b(s_{i},t_{n})}{60h}(Z_{i+1}-Z_{i-1})} +{\frac{b(s_{i+1},t_{n})}{60h}}(Z_{i-1}-4Z_{i}+3Z_{i+1}) + {\frac{P(s_{i-1},t_{n})}{30}}Z_{i-1}\\ {}&+{\frac{28P(s_{i},t_{n})}{30}Z_{i}} +{\frac{P(s_{i+1},t_{n})}{30}Z_{i+1}} = {\frac{1}{30}}(R(s_{i-1},t_{n}) +28R(s_{i},t_{n})+R(s_{i+1},t_{n})). \end{aligned}$$

#### Computing the exponential fitting factor

In this part, we introduce the fitting factor $\sigma$ and for the obtained scheme of (6)–(7) at (*i*, *n*)*th* level. As the theory of singular perturbation given in [9, 23], the zero order asymptotic solution of the problem of the form

17 $${\left\{ \begin{array}{ll} -\varepsilon Z^{''}(s) +b(s)Z^{'}+p(s)Z(s) =q(s),~~~ s\in \Omega _{s} = (0,1),\\ Z(0) = \alpha _{l},~~~Z(1) = \alpha _{r}, \end{array}\right. }$$

is given by

18 $$\begin{aligned} Z(s) \approx Z_{0}(s) + \frac{b(0)}{b(s)}(\alpha _{l} - Z_{0}(0))\exp\bigg(-{\int _{0}^{s}\left(\frac{b(s)}{\varepsilon }-\frac{p(s)}{b(s)}\right)ds\bigg) + O(\varepsilon )}. \end{aligned}$$

From Taylor’s series expansion for *b*(*s*) and *p*(*s*) restricting to their first terms about $s =0$ and the simplified form gives

19 $$\begin{aligned} Z(s) = Z_{0}(s)+(\alpha _{l}-Z_{0}(0))\exp \left(-\frac{b(0)}{\varepsilon }s\right), \end{aligned}$$

where $Z_{0}$ is the reducible problem solution. Considering *h* is fairly small and solving the result in (19) at $s_{i}$ which gives

$$\begin{aligned} Z(s_{i}) =Z(ih) = Z_{0}(0)+(\alpha _{l}-Z_{0}(0))\exp (-b(0)i\rho ), \end{aligned}$$

where $\rho = \frac{h}{\varepsilon }.$ We present an exponentially fitting factor $\sigma$ of the scheme (16)

20 $$\begin{aligned}&-{\left(\varepsilon \sigma -{\frac{hb(s_{i-1},t_{n})}{30}}+{\frac{hb(s_{i+1},t_{n})}{30}}\right)}\left({\frac{Z_{i-1} - 2Z_{i} + Z_{i+1}}{h^2}}\right) + {\frac{b(s_{i-1},t_{n})}{60h}}(-3Z_{i-1}+4Z_{i}-Z_{i+1})\\&+{\frac{28b(s_{i},t_{n})}{60h}}(Z_{i+1}-Z_{i-1}) +{\frac{b(s_{i+1},t_{n})}{60h}}(Z_{i-1}-4Z_{i}+3Z_{i+1}) + {\frac{P(s_{i-1},t_{n})}{30}}Z_{i-1}\\ {}&+{\frac{28P(s_{i},t_{n})}{30}}Z_{i}+{\frac{P(s_{i+1},t_{n})}{30}}Z_{i+1} = {\frac{1}{30}}(R(s_{i-1},t_{n}) +28R(s_{i},t_{n})+R(s_{i+1},t_{n})). \end{aligned}$$

Putting $\rho = \frac{h}{\varepsilon }$ and after multiplying both side by *h* and letting $h\rightarrow 0$ which gives

21 $$\begin{aligned}&-{\frac{\sigma }{\rho }}\lim _{h \rightarrow 0} (Z_{i-1} -2Z_{i}+Z_{i+1}) + {\frac{b_{0}(t_{n})}{60}}\lim _{h \rightarrow 0}(-3Z_{i-1}+4Z_{i}-Z_{i+1}) \\&+{\frac{28b_{0}(t_{n})}{60}}\lim _{h \rightarrow 0}(Z_{i+1}-Z_{i-1}) +{\frac{b_{0}(t_{n})}{60}}\lim _{h \rightarrow 0}(Z_{i-1}-4Z_{i}+3Z_{i+1}) = 0. \end{aligned}$$

Using the results in (19) and simplifying, we obtain

22 $$\begin{aligned} \frac{\sigma }{\rho }(e^{ b(0)\rho } + e^{-b(0)\rho } - 2) = \frac{b_0(t_{n})}{60}(-30e^{b(0)\rho }+30e^{-b(0)\rho }). \end{aligned}$$

Solving for $\sigma$ in (22), the exponential fitting factor $\sigma$ is obtained as

23 $$\begin{aligned} \sigma = \frac{\rho b_0(t_{n})}{2}\coth \left(\frac{\rho b(0)}{2}\right). \end{aligned}$$

### The discrete scheme

Using the higher order finite difference scheme of (16) and inducing the exponential fitting factor (23) for $1\le i \le N-1$ and $0 \le n \le M-1$, then the fully discrete scheme is given as

24 $$\begin{aligned} L^{\Delta t,h}Z_{i}^{n}= \frac{1}{30}(R(s_{i-1},t_{n}) +28R(s_{i},t_{n})+R(s_{i+1},t_{n})), \end{aligned}$$

where $$L^{\Delta t,h}Z_{i}^{n}=-(\varepsilon \sigma -\frac{hb(s_{i-1},t_{n})}{30}+\frac{hb(s_{i+1},t_{n})}{30})(\frac{Z_{i-1}^{n} - 2Z_{i}^{n} + Z_{i+1}^{n}}{h^2}) + \frac{b(s_{i-1},t_{n})}{60h}(-3Z_{i-1}^{n}+4Z_{i}^{n}-Z_{i+1}^{n}) +\frac{28b(s_{i},t_{n})}{60h}(Z_{i+1}^{n}-Z_{i-1}^{n}) +\frac{b(s_{i+1},t_{n})}{60h}(Z_{i-1}^{n}-4Z_{i}^{n}+3Z_{i+1}^{n}) + \frac{P(s_{i-1},t_{n})}{30}Z_{i-1}^{n}+\frac{28P(x_{i},t_{n})}{30}Z_{i}^{n} +\frac{P(s_{i+1},t_{n})}{30}Z_{i+1}^{n}.$$

In the explicit form, we write

25 $$\begin{aligned} r_{i}^{-}Z_{i-1}^{n}+r_{i}^{0}Z_{i}^{n}+r_{i}^{+}Z_{i+1}^{n} = H_{i}^{n}, \end{aligned}$$

where

26 $$\begin{aligned} \begin{aligned} r_{i}^{-} =&-\frac{1}{h^2}\left(\varepsilon \sigma - \frac{hb(s_{i-1},t_{n})}{30} +\frac{hb(s_{i+1},t_{n})}{30}\right) - \frac{3b(s_{i-1},t_{n})}{60h}+\frac{P(s_{i-1},t_{n})}{30}\\&-\frac{28b(s_{i},t_{n})}{60h}+\frac{b(s_{i+1},t_{n})}{60h}, \\ r_{i}^{0} =&\frac{2}{h^2}\left(\varepsilon \sigma - \frac{hb(s_{i-1},t_{n})}{30} +\frac{hb(s_{i+1},t_{n})}{30}\right) + \frac{4b(s_{i-1},t_{n})}{60h}-\frac{4b(s_{i+1},t_{n})}{60h}+\frac{28P(s_{i},t_{n})}{30}, \\ r_{i}^{+} =&-\frac{1}{h^2}\left(\varepsilon \sigma - \frac{hb(s_{i-1},t_{n})}{30} +\frac{hb(s_{i+1},t_{n})}{30}\right) - \frac{b(s_{i-1},t_{n})}{60h}+\frac{28b(s_{i},t_{n})}{60h}\\ {}&+\frac{3b(s_{i+1},t_{n})}{60h} +\frac{P(s_{i+1},t_{n})}{30}, \\ H_{i}^{n} =&\frac{1}{30}(R(s_{i-1},t_{n}) +28R(s_{i},t_{n})+R(s_{i+1},t_{n})). \end{aligned} \end{aligned}$$

### Stability and convergence analysis

In this part, for the developed scheme of (24) we take to prove the discrete comparison principle.

#### Lemma 3.3

*There is a comparison function* $\nu _{i}^{n}$ *such that* ${\mathcal {L}}^{\Delta t,h}Z_{i}^{n} \le {\mathcal {L}}^{\Delta t,h}\nu _{i}^{n}$ *for* $1 \le i \le N-1$ *and if* $Z_{0}^{n}\le \nu _{0}^{n}$ *and* $Z_{N}^{n}\le \nu _{N}^{n}$, *then*  $Z_{i}^{n} \le \nu _{i}^{n}$ *for* $1 \le i\le N$.

#### Proof

The discrete operator ${\mathcal {L}}^{\Delta t,h}Z_{i}^{n}$ is matrix of size $(N+1) \times (N+1)$ with its entries for $1\le i \le N-1$ are $r_{i}^{-}, \;r_{i}^{0},$ and $r_{i}^{+}.$ We observe that $\vert r_{i}^{-} |> 0, \ \vert r_{i}^{0} |> 0, \ \vert r_{i}^{+} |>0$ and $\vert r_{i}^{0} |\ge \vert r_{i}^{-} |+ \vert r_{i}^{+} |$, giving that the matrix is diagonally dominant. Then, it satisfies the property of *M* matrix. Thus, the non-negative inverse of the matrix exists. So, it guarantees the existence of unique discrete solution [22, 24]. $\square$

#### Lemma 3.4

*(Stability result) If the solution of (*24*) be* $Z_{i}^{n}$, *then it satisfies*

$$\begin{aligned} \big \vert Z_{i}^{n}\big |\le \frac{\Vert \ {\mathcal {L}}^{\Delta t,h}Z_{i}^{n}\Vert }{ \zeta } + \max \{\vert \psi _{l}(t_{n})\vert ,\vert \psi _{r}(t_{n})|\}, \end{aligned}$$

*where* $$P(s_{i},t_{n}) \ge \zeta > 0$$.

#### Proof

let $\Pi = \frac{\Vert \ {\mathcal {L}}^{\Delta t,h}Z_{i}^{n}\Vert }{ \zeta } + \max \{\vert \psi _{l}(t_{n})\vert ,\vert \psi _{r}(t_{n})|\}$ and set the barrier functions $\vartheta _{i,n}^{\pm }$ by $\vartheta _{i,n}^{\pm } = \Pi \pm Z_{i}^{n}$. On the boundaries, we obtain $\vartheta _{0,n}^{\pm } = \Pi \pm Z_{0}^{n} = \frac{\Vert \ {\mathcal {L}}^{\Delta t,h}Z_{i}^{n}\Vert }{ \zeta } + \max \{\vert \psi _{l}(t_{n})\vert ,\vert \psi _{r}(t_{n})|\} \pm \psi _{l}(t_{n}) \ge 0$,

$\vartheta _{N,n}^{\pm } = \Pi \pm Z_{N}^{n} = \frac{\Vert \ {\mathcal {L}}^{\Delta t,h}Z_{i}^{n}\Vert }{ \zeta } + \max \{\vert \psi _{l}(t_{n})\vert ,\vert \psi _{r}(t_{n})|\} \pm \psi _{r}(t_{n}) \ge 0$. On the discretized spatial domain $s_{i} , 1< i < N-1,$ we have

27 $$\begin{aligned} \begin{aligned} {\mathcal {L}}^{\Delta t,h}\vartheta _{i,n} =&- \left(\varepsilon \sigma -\frac{hb(s_{i-1},t_{n})}{30}+\frac{hb(s_{i+1},t_{n})}{30}\right)\left(\frac{\Pi \pm Z_{i-1}^{n} - 2(\Pi \pm Z_{i}^{n}) + \Pi \pm Z_{i+1}^{n}}{h^2}\right)\\&+ \frac{ b(s_{i-1},t_{n})}{60h}(-3(\Pi \pm Z_{i-1}^{n})+4(\Pi \pm Z_{i}^{n})-(\Pi \pm Z_{i+1}^{n})) \\ {}&+\frac{28b(s_{i},t_{n})}{60h}(\Pi \pm Z_{i+1}^{n}-(\Pi \pm Z_{i-1}^{n})) \\&+\frac{b(s_{i+1},t_{n})}{60h}(\Pi \pm Z_{i-1}^{n}-4(\Pi \pm Z_{i}^{n})+3(\Pi \pm Z_{i+1}^{n})) \\&+ \frac{P(s_{i-1},t_{n})}{30}(\Pi \pm Z_{i-1}^{n}) +\frac{28P(s_{i},t_{n})}{30}(\Pi \pm Z_{i}^{n}) +\frac{P(s_{i+1},t_{n})}{30}(\Pi \pm Z_{i+1}^{n}) \\ =&\left(\frac{P(s_{i-1},t_{n})}{30} +\frac{28P(s_{i},t_{n})}{30} +\frac{P(s_{i+1},t_{n})}{30}\right)\Pi \pm {\mathcal {L}}^{\Delta t,h}Z_{i}^{n}\\ =&\left(\frac{P(s_{i-1},t_{n})}{30} +\frac{28P(s_{i},t_{n})}{30} +\frac{P(s_{i+1},t_{n})}{30}\right)\left(\frac{\Vert \ {\mathcal {L}}^{\Delta t,h}Z_{i}^{n}\Vert }{ \zeta } + \max \{\vert \psi _{l}(t_{n})\vert ,\vert \psi _{r}(t_{n})|\}\right) \pm \frac{1}{30}(R_{i-1}^{n} +28R_{i}^{n} +R_{i+1}^{n})\ge 0. \end{aligned} \end{aligned}$$

From the discrete comparison principle, we get $\vartheta _{i,n}^{\pm } \ge 0,\ i = 0,1,2,...,N$. Hence, the necessary bound is satisfied. $\square$

#### Lemma 3.5

*If* $\nu _{i}^{n}$ *be any mesh function such that* $\nu _{0}^{n} = \nu _{N}^{n} = 0$. *Then it satisfies*

28 $$\begin{aligned} \big \vert \nu _{i}^{n}\big |\le \frac{1}{\zeta }\max _{m}\big \vert {\mathcal {L}}^{\Delta t, h}\nu _{m}^{n}\big |. \end{aligned}$$

Using Taylor’s series approximation, we have the bounds

29 $$\begin{aligned} \begin{aligned} \bigg \vert - (\frac{d}{ds^2} - \delta ^{2}_{s})Z^{n}(s_i)\bigg |\le&Ch^{2} \bigg \Vert \frac{d^{4}Z^{n}(s_i)}{ds^4}\bigg \Vert ,\\ \bigg \vert \frac{dZ^{n}(s_{i-1})}{ds} -(\frac{-Z_{i+1}^{n} +4Z_{i}^{n}- 3Z_{i-1}^{n}}{2h} + h\frac{d^{2}Z^{n}(s_{i})}{ds^{2}}) \bigg |\le&Ch^{2} \bigg \Vert \frac{d^{3}Z^{n}(s_i)}{ds^3}\bigg \Vert ,\\ \bigg \vert \frac{dZ^{n}(s_{i+1})}{ds} -(\frac{3Z_{i+1}^{n} -4Z_{i}^{n}+ Z_{i-1}^{n}}{2h} - h\frac{d^{2}Z^{n}(s_{i})}{ds^{2}}) \bigg |\le&Ch^{2}\bigg \Vert \frac{d^{3}Z^{n}(s_i)}{ds^3}\bigg \Vert ,\\ \bigg \vert (\frac{d}{ds}-\delta ^{0}_{s})Z^{n}(s_i)\bigg |\le Ch^{2} \bigg \Vert \frac{d^{3}Z^{n}(s_i)}{ds^3}\bigg \Vert , \bigg \vert \delta ^{2}_{s}Z^{n}(s_i) \bigg |\le&C\bigg \Vert \frac{d^{2}Z^{n}(s_i)}{ds^{2}}\bigg \Vert , \end{aligned} \end{aligned}$$

where $$\Vert Z^{(k)}(s_i)\Vert = \max _{s_{i}}\vert Z_{n}^{(k)}(s_i) |\ , k = 2, 3, 4$$.

In the next theorem we bound the truncation error in space direction discretization.

#### Theorem 3.1

*Consider the sufficiently smooth functions* $a(s), \ b(s,t_{n})$ *and* $c(s,t_{n})$ *of (*6–7) *so that* $Z^{n}(s)\in C^{4}[0,1]$. *Then, the solution* $Z_{i}^{n}$ *of (*24) *satisfies the bound*

30 $$\begin{aligned} \big \vert {\mathcal {L}}^{\Delta t, h}(Z^{n}(s_{i})-Z_{i}^{n})\big |\le \frac{Ch^{2}}{h+\varepsilon }\left(1+\varepsilon ^{-3}\exp \left(-\frac{\beta }{\varepsilon }s_{i}\right)\right). \end{aligned}$$

#### Proof

In the spatial direction the local truncation error is given by

$$\begin{aligned} \begin{aligned} \bigg \vert {\mathcal {L}}^{\Delta t, h}(Z^{n}(s_{i})&-Z_{i}^{n})\bigg |= \bigg \vert - \varepsilon \left(\frac{d}{ds^2} -\sigma \delta ^{2}_{s}\right)Z^{n}(s_i) + \frac{ b(s_{i-1},t_{n})}{60h}\\ {}&\left(\frac{dZ^{n}(s_{i-1})}{ds} -\left(\frac{-Z_{i+1}^{n} +4Z_{i}^{n}- 3Z_{i-1}^{n}}{2h} + h\frac{d^{2}Z^{n}(s_{i})}{ds^{2}}\right)\right) \\&+\frac{28b(s_{i},t_{n})}{60h}\left(\frac{d}{ds}-\delta ^{0}_{s}\right)Z^{n}(s_i) +\frac{b(s_{i+1},t_{n})}{60h}\\ {}&+ \left(\frac{dZ^{n}(s_{i+1})}{ds}-\left(\frac{3Z_{i+1}^{n} -4Z_{i}^{n}+ Z_{i-1}^{n}}{2h} - h\frac{d^{2}Z^{n}(s_{i})}{ds^{2}}\right)\right)\bigg |\\ \le&\bigg \vert \left(\varepsilon (b(s_{i},t_{n})\frac{\rho }{2}\coth \left(b(0)\frac{\rho }{2}\right) -1\right)\delta ^{2}_{s}Z^{n}(s_i))\bigg |+\bigg \vert \varepsilon \left(\frac{d^2}{ds^2}-\delta ^{2}_{s}\right)Z^{n}(s_{i})\bigg |\\ {}& \bigg \vert \frac{ b(s_{i-1},t_{n})}{60h}(\frac{dZ^{n}(s_{i-1})}{d s}-(\frac{-Z_{i+1}^{n} +4Z_{i}^{n}- 3Z_{i-1}^{n}}{2h} + h\frac{d^{2}Z^{n}(s_{i})}{d s^{2}}))\bigg |\\&+\bigg \vert \frac{28b(s_{i},t_{n})}{60h}\left(\frac{d}{ds}-\delta ^{0}_{s}\right)Z^{n}(s_i) \bigg |+\bigg \vert \frac{b(s_{i+1},t_{n})}{60h}\\ {}&\left(\frac{d Z^{n}(s_{i+1})}{ds}-\left(\frac{3Z_{i+1}^{n} -4Z_{i}^{n}+ Z_{i-1}^{n}}{2h} - h\frac{d^{2}Z^{n}(s_{i})}{ds^{2}}\right)\right) \bigg |, \end{aligned} \end{aligned}$$

where $$\sigma = b(s_{i},t_{n})\frac{\rho }{2}\coth (b(0)\frac{\rho }{2}) \ and \ \rho = \frac{h}{\varepsilon }$$.

For the constants $C_{1}$ and $C_{2}$ we have $\big \vert \rho \coth (\rho )-1\big |\le C_{1}\rho ^{2}$, for $\rho \le 1$. For $\rho \rightarrow \infty$, since $\lim _{\rho \rightarrow \infty }\coth (\rho )=1$ which gives $\big \vert \rho \coth (\rho )-1\big |\le C_{1}\rho$.

Generally, $\forall \rho > 0$, we put as

31 $$\begin{aligned} C_{1}\frac{\rho ^{2}}{\rho +1}\le \rho \coth (\rho )-1\le C_{2}\frac{\rho ^{2}}{\rho +1}, \end{aligned}$$

we obtain

32 $$\begin{aligned} \varepsilon [b(s_{i},t_{n})\frac{\rho }{2}\coth \left(b(0)\frac{\rho }{2}\right)-1]\le \varepsilon \frac{(h/\varepsilon )^{2}}{h/\varepsilon +1} = \frac{h^2}{h+\varepsilon }. \end{aligned}$$

Using the bound for the difference of the derivatives in (29) and (32), we obtain

$$\begin{aligned} \bigg \vert {\mathcal {L}}^{\Delta t, h}(Z^{n}(s_{i})-Z_{i}^{n})\bigg \vert \le \frac{Ch^2}{h+\varepsilon } \bigg \Vert \frac{d^{2}Z^{n}(s_i)}{ds^{2}}\bigg \Vert +Ch^{2}\bigg \Vert \frac{d^{3}Z^{n}(s_i)}{ds^3}\bigg \Vert + C\varepsilon h^{2}\bigg \Vert \frac{d^{4}Z^{n}(s_{i})}{ds^{4}}\bigg \Vert . \end{aligned}$$

From Lemma 3.2, we obtain the bound for the derivatives

33 $$\begin{aligned} \begin{aligned} \bigg \vert {\mathcal {L}}^{\Delta t, h}(Z^{n}(s_{i})-Z_{i}^{n})\bigg \vert \le&\frac{Ch^2}{h+\varepsilon }\left(1+\varepsilon ^{-2}\exp \left(-\frac{\beta }{\varepsilon }s_{i}\right)\right) +Ch^{2}\left[\left(1+\varepsilon ^{-3}\exp \left(-\frac{\beta }{\varepsilon }s_{i}\right)\right)+ \left(\varepsilon +\varepsilon ^{-3}\exp \left(-\frac{\beta }{\varepsilon }s_{i}\right)\right)\right]. \end{aligned} \end{aligned}$$

Evidently, $\varepsilon ^{-2} \le \varepsilon ^{-3}$, then we obtain $\big \vert {\mathcal {L}}^{\Delta t, h}(Z^{n}(s_{i})-Z_{i}^{n})\big |\le \frac{Ch^2}{h+\varepsilon }(1+\varepsilon ^{-3}\exp (-\frac{\beta }{\varepsilon }s_{i}))$ thus, it gives the desired bound. $\square$

#### Lemma 3.6

*For a fixed mesh and as* $\varepsilon \rightarrow 0,$* it gives*

34 $$\begin{aligned} \lim _{\varepsilon \rightarrow 0}\max _{i}\frac{\exp (-\beta s_{i}/\varepsilon )}{\varepsilon ^n} = 0, \ n = 1,2,3,..., \end{aligned}$$

*where* $$s_{i} = ih, \ 1 \le i \le N-1$$.

#### Proof

The proof is considered in [9]. $\square$

#### Theorem 3.2

*Let* $Z_{i}^{n}$* be the solution of (*24),* then we have the following uniform error bound*

35 $$\begin{aligned} \sup _{\varepsilon \in (0, 1]}\max _{i}\big \vert Z^{n}(s_{i})-Z_{i}^{n}\big |\le Ch, \ i = 0,1,2,...N. \end{aligned}$$

#### Proof

Plugging the result in Lemma 3.6 into (30), we arrive at

36 $$\begin{aligned} \bigg \vert {\mathcal {L}}^{\Delta t, h}(Z^{n}(s_{i})-Z_{i}^{n})\bigg |\le \frac{Ch^2}{h+\varepsilon }. \end{aligned}$$

Hence, the result leads $\big \vert Z^{n}(s_i)-Z_{i}^{n}\big |\le \frac{Ch^2}{h+\varepsilon }.$ Using the $\sup$ over all $\varepsilon \in (0,1]$, we get

37 $$\begin{aligned} \sup _{\varepsilon \in (0, 1]} \max _{i}\big \vert Z^{n}(s_{i})-Z_{i}^{n}\big |\le Ch. \end{aligned}$$

From the preceding theorem for the case when $\varepsilon > h$, the obtained scheme gives second order uniformly convergent. For the case when $\varepsilon \ll h$, the scheme is first order uniformly convergent in spatial direction. $\square$

#### Theorem 3.3

*Let* *z and* *Z are the solutions of (*1*) and (*24) *respectively, then we have the following uniform error bound*

38 $$\begin{aligned} \sup _{\varepsilon \in (0, 1]}\big \vert z-Z \big |\le C(h+(\Delta t)). \end{aligned}$$

#### Proof

The proof can be done by the combination of Lemma 3.1 and Theorem 3.2. $\square$

## Numerical results and discussions

In this part, we are considering two model examples to validate the theoretical results obtained by the proposed method. If the exact solutions of the considered examples are not known the maximum pointwise error is estimated by using the double mesh principle. So, the maximum pointwise error is calculated by $E_{\varepsilon }^{N,M} = \max _{i,n}\vert Z_{i,n}^{N,M}- Z_{i,n}^{2N,2M}|,$ and the $\varepsilon$-uniform error is estimated by $E^{N,M} = \max _{i,n}(E_{\varepsilon }^{N,M}).$ The rate of convergence is calculated by $r^{N,M}_{\varepsilon } = \log 2(E^{N,M}_{\varepsilon }/E^{2N,2M}_{\varepsilon }),$ and the $\varepsilon$-uniform rate of convergence is computed by $r^{N,M} = \log 2(E^{N,M}/E^{2N,2M}).$

### Example 4.1

Consider the problem $\frac{\partial u}{\partial t} - \varepsilon \frac{\partial ^2u}{\partial x^2} - \frac{\partial u}{\partial x}+(\frac{1+x^2}{2})u = t^3 - u(x,t-\tau ),~~(x,t)\in (0,1)\times (0,2]$ with interval condition u(x,t) = 0, on $(x,t) \in [0,1]\times [-\tau ,0]$ and the boundary conditions $u(0,t) = 0~~ and ~~u(1,t) = 0,~~~t\in (0,2].$

### Example 4.2

Consider the problem $\frac{\partial u}{\partial t} - \varepsilon \frac{\partial ^2u}{\partial x^2} -(2+x^{2}) \frac{\partial u}{\partial x}+xu(x,t) + u(x,t-\tau ) = 10t^{2}\exp (-t)x(1-x),$

$$(x,t)\in (0,1)\times (0,2]$$

with interval condition $u(x,t) = 0$, on $(x,t) \in [0,1]\times [-\tau ,0]$ and the boundary conditions $u(0,t) = 0~~ and ~~u(1,t) = 0,~~~t\in (0,2].$

**Fig. 1:**
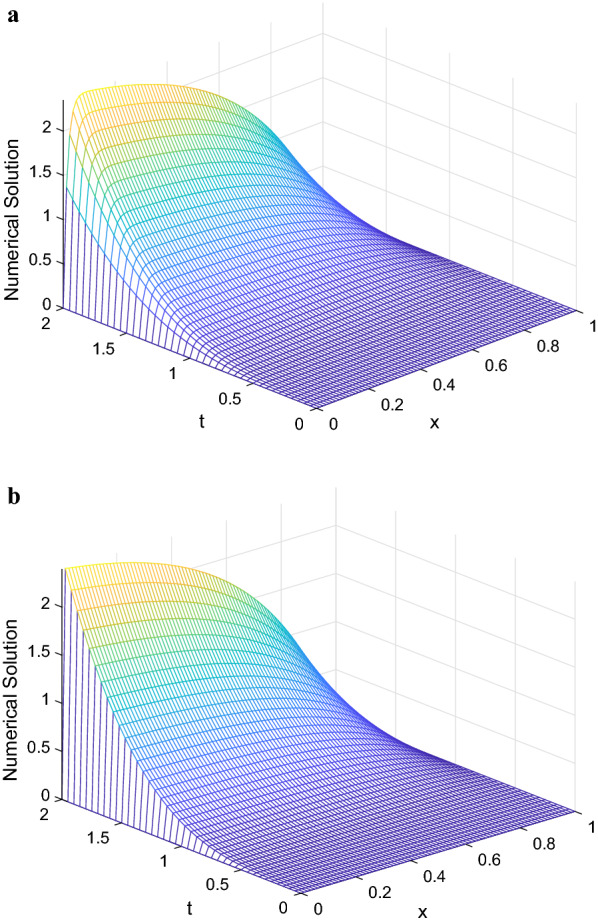
Numerical solution of Example 4.1 on a, $\varepsilon = 2^{-6}$ and b, $\varepsilon = 2^{-20}$

**Table 1:** Example 4.1, maximum absolute errors for the case $\tau = 0.5\varepsilon$

$\varepsilon \downarrow$	N = M =16	32	64	128	256
$2^{-0}$	1.3260e−03	7.4390e−04	3.9431e−04	2.0293e−04	1.0294e−04
$2^{-2}$	1.3097e−02	7.2784e−03	3.8340e−03	1.9676e−03	9.9661e−04
$2^{-4}$	2.7648e−02	1.5726e−02	8.3824e−03	4.3310e−03	2.2011e−03
$2^{-6}$	3.7674e−02	1.9780e−02	1.0209e−02	5.3769e−03	2.7671e−03
$2^{-8}$	3.7154e−02	2.2029e−02	1.2040e−02	5.6488e−03	2.8289e−03
$2^{-10}$	3.7206e−02	2.1942e−02	1.1903e−02	6.2248e−03	3.1986e−03
$2^{-12}$	3.7221e−02	2.1951e−02	1.1906e−02	6.1986e−03	3.1627e−03
$2^{-14}$	3.7224e−02	2.1953e−02	1.1907e−02	6.1992e−03	3.1627e−03
$2^{-16}$	3.7225e−02	2.1953e−02	1.1907e−02	6.1993e−03	3.1627e−03
$2^{-18}$	3.7225e−02	2.1953e−02	1.1908e−02	6.1994e−03	3.1627e−03
$2^{-20}$	3.7225e−02	2.1953e−02	1.1908e−02	6.1994e−03	3.1627e−03
Proposed scheme					
$E^{N,M}$	3.7225e−02	2.2029e−02	1.2040e−02	6.2248e−03	3.1986e−03
$r^{N,M}$	0.77417	0.87156	0.95174	0.96059	–
Method in [14]					
$E^{N,M}$	8.3951e−02	4.9224e−02	2.6666e−02	1.3880e−02	7.0816e−03
$r^{N,M}$	0.77019	0.88436	0.94199	0.97086	–

**Table 2:** Example 4.2, maximum absolute errors for the case $\tau = 0.5\varepsilon$

$\varepsilon \downarrow$	N = M =16	32	64	128	256
$2^{-0}$	2.4094e−04	4.5191e−05	5.3745e−06	4.6776e−06	3.4511e−06
$2^{-2}$	5.8848e−04	1.7058e−04	1.1039e−04	6.2476e−05	3.3191e−05
$2^{-4}$	2.1117e−03	7.2963e−04	3.0344e−04	1.3611e−04	6.4377e−05
$2^{-6}$	4.6754e−03	2.2578e−03	8.2105e−04	2.8430e−04	1.0734e−04
$2^{-8}$	4.8645e−03	2.9411e−03	1.5511e−03	6.7433e−04	2.3656e−04
$2^{-10}$	4.8682e−03	2.9448e−03	1.6029e−03	8.4404e−04	4.2558e−04
$2^{-12}$	4.8691e−03	2.9452e−03	1.6032e−03	8.4463e−04	4.3880e−04
$2^{-14}$	4.8693e−03	2.9453e−03	1.6032e−03	8.4466e−04	4.3881e−04
$2^{-16}$	4.8693e−03	2.9454e−03	1.6032e−03	8.4467e−04	4.3881e−04
$2^{-18}$	4.8694e−03	2.9454e−03	1.6032e−03	8.4467e−04	4.3881e−04
$2^{-20}$	4.8694e−03	2.9454e−03	1.6032e−03	8.4467e−04	4.3881e−04
$E^{N,M}$	4.8694e−03	2.9454e−03	1.6032e−03	8.4467e−04	4.3881e−04
$r^{N,M}$	0.7253	0.8775	0.9245	0.9448	–

For different values of $\varepsilon$ and an equal number of mesh points the maximum pointwise error, $\varepsilon$ -uniform error and $\varepsilon$-uniform rate of convergence of the proposed method are displayed in Tables 1 and 2. We observe from these Tables, as $\varepsilon \rightarrow 0$, the maximum pointwise error after showing increment remains uniform. This shows that the scheme is stable and uniformly convergent irrespective of the values of $\varepsilon$. The $\varepsilon$-uniform error and $\varepsilon$-uniform rate of convergence of the method are indicated in the last rows of each Tables and it confirms that the numerical results agree with the theoretical result.

In Fig. 1, we show the numerical solution of the scheme for Example 4.1 for different values of $\varepsilon$. From these Figures, it can be seen that a strong boundary layer is created on the left side of the spatial domain for small $\varepsilon$.

In the last section of Table 1, the comparison of the proposed scheme with the results of the existing published work of [14] is given. As one observes, the developed scheme gives more accurate result than the scheme in [14].

## Conclusion

We developed a numerical scheme to solve a singularly perturbed parabolic convection-diffusion equation that exhibits a boundary layer. The proposed scheme consists of the backward-Euler method in the time direction and an exponentially fitted finite difference scheme for the spatial direction. Using the comparison principle, the stability of the discrete scheme is examined and analysed. The uniform convergence of the scheme is discussed theoretically. To validate the theoretical finding of the scheme, we considered two model examples and the numerical results are given by applying maximum point-wise absolute error, $\varepsilon$-uniform error and $\varepsilon$-uniform rate of convergence in Tables. The proposed method contributes more accurate, stable and $\varepsilon$-uniform numerical result with linear order of convergence.

## Limitations

- The proposed scheme is not layer resolving method since there is no sufficient number of mesh points in the boundary layer region.

## Data Availability

No additional data is used for this research work.

## Appendix

Proof of Lemma 3.1 Since the function $Z^{n}(s)$ satisfies

$$\begin{aligned}&(1-\tau c(s,t_{n}) + \Delta t {\mathcal {L}})Z^{n}(s) - \Delta g(s, t_{n}) = Z^{n-1}(s)\\ Z(t_{n-1}) =&(1-\tau c(t_n)+\Delta t {\mathcal {L}})Z(t_n) -\Delta g(t_n)+ \int _{t_{n-1}}^{t_n}(t_{n-1}-s){\frac{\partial ^{2}Z(s)}{\partial t^2}}ds\\ =&(1-\tau c(t_n)+\Delta t {\mathcal {L}})Z(t_n) -\Delta g(t_n) +O(\Delta t^2). \end{aligned}$$

Then $e_{n}(s)$ is the solution of boundary value problem of type

$$((1-\tau c(s,t_n)) +\Delta t {\mathcal {L}})e_{n}(s) = O(\Delta t^2)$$.

$e_{n}(0) = 0 = e_{n}(1)$ by manimum principle, we obtain $\Vert e_{n} \Vert \le C_{1}(\Delta t^2)$.

Taking the summation of all local error estimate up to $n^{th}$ time step the global error estimate is given by

$$\begin{aligned} \begin{aligned} \Vert E_{n}\Vert =&\bigg \Vert \sum _{l=1}^{n}e_{l}\bigg \Vert \le \Vert e_{1}\Vert + \Vert e_{2}\Vert + \Vert e_{3} \Vert + . . . + \Vert e_{n}\Vert \\ \le&C_{1}T(\Delta t) , ~~since~~ (n)\Delta t \le T \\ =&C(\Delta t), ~~~ where~~ C_{1}T = C, \end{aligned} \end{aligned}$$

where *C* is a constant not depends on $\varepsilon$ and $\Delta t$.
